# Ultrasound-Guided Regional Anesthesia as Primary Analgesic Management in the Orthopedic-Surgical Emergency Department of an Affiliated Hospital: A Retrospective Analysis over a 6-Year Period

**DOI:** 10.3390/medicina61112006

**Published:** 2025-11-10

**Authors:** Eckehart Schöll, Mark Ulrich Gerbershagen, Andreas Marc Müller, Rainer Jürgen Litz

**Affiliations:** 1 Department of Emergency Medicine, Bethesda Spital, 4052 Basel, Switzerland; 2 Faculty of Medicine, University of Witten Herdecke, 58313 Herdecke, Germany; 3 Department of Anaesthesiology, Hospital Cologne Holweide, 51067 Cologne, Germany; 4 Department of Orthopedics, University Hospital Basel, 4031 Basel, Switzerland; a.mueller@usb.ch; 5 Independent Researcher, 86199 Augsburg, Germany; r.j.litz@usra.de

**Keywords:** ultrasound-guided regional anesthesia, emergency medicine, peripheral nerve blocks, pain management, orthopedic-trauma emergencies

## Abstract

*Background and Objectives*: Ultrasound (US)-guided peripheral regional anesthesia (pRA) is gaining increasing importance in emergency medicine as an effective, low-ridsk alternative to general anesthesia (GA), procedural sedation (PS), or opioid therapy. By enabling rapid, direct pain management in the emergency department (ED), pRA can help preserve scarce surgical and anesthetic resources and, in some cases, avoid inpatient admissions. The aim of this study was to analyze the indications, techniques, and clinical impact of pRA in the orthopedic-focused ED of an affiliated hospital. *Materials and Methods*: All pRA and PS procedures performed over a six-year period were retrospectively reviewed among 35,443 orthopedic-trauma emergency patients. pRA was carried out under US guidance with standardized monitoring. Diagnoses, block techniques, effectiveness, and complications were analyzed descriptively. *Results*: A total of 1292 patients (3.7%) underwent either pRA (*n* = 1117; 3.2%) or PS (*n* = 175; 0.5%). pRA was performed in 22% of cases for interventions such as reductions or extensive wound management. In 78%, pRA was applied for analgesia, for example, in the diagnostic work-up and treatment of non-immediately operable fractures, lumbago, or arthralgia. The most common pRA techniques were brachial plexus blocks (54%) and femoral nerve blocks (25%). Fascial plane blocks (6.1%) and paravertebral blocks (1.5%) were rarely used. PS was performed in 175 of 1292 patients (13%), although pRA would have been feasible in 159 of these cases. No complications of pRA were observed, and GA could routinely be avoided. *Conclusions*: US-guided pRA proved to be an effective and safe alternative to PS, GA, or systemic analgesia for selected indications, allowing immediate treatment without the need for operative capacities. To ensure safe application, these techniques should be an integral part of the training curriculum for ED personnel.

## 1. Introduction

Among the most common reasons for presentation to an orthopedic emergency department (ED) are musculoskeletal injuries, soft-tissue infections, acute low-back or cervical pain, and exacerbated arthralgia. In many cases, management can be provided directly in ED procedure rooms, outside of the regular operating theater workflow, and without the need for surgical or inpatient capacities. However, such injuries and conditions usually require immediate and effective pain control. Initial analgesia can often be achieved by parenteral or enteral administration of analgesics; however, this is frequently inadequate to allow effective and timely management of the underlying condition within the ED. Minor surgical procedures may be performed under infiltration or local anesthesia (LA) by ED physicians themselves. For more painful or extensive procedures, such as reductions or treatment of large wounds and infections, infiltration anesthesia is usually insufficient. In these situations, procedural sedation (PS) or even general anesthesia (GA) administered by anesthesiologists is often required.

**Figure 1:**
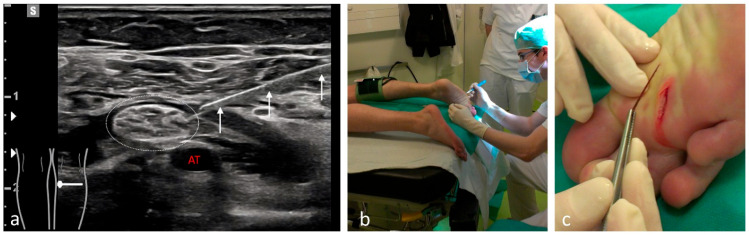
Removal of a deeply embedded splinter from the plantar surface under pRA with a tourniquet. (**a**) The three white arrows indicate the needle during tibial nerve anesthesia (white dotted oval) located above the tibial artery (AT). (**b**) During the procedure, the awake patient is placed in the prone position with a tourniquet applied to the lower leg. (**c**) The splinter can be removed without difficulty under complete tourniquet control, allowing full exploration of the wound.

The integration of emergency patients into the scheduled surgical program during core working hours frequently leads to delays due to limited operating room (OR) and anesthetic capacities. Outside of core hours, these resources are often reserved for cases of higher priority. The resulting long waiting times can be burdensome for ED patients and negatively affect patient satisfaction and treatment outcomes [1,2]. For years, peripheral regional anesthesia (pRA) has, therefore, been performed in EDs in many countries, as well as by surgeons or emergency physicians themselves [3,4,5], generally allowing for faster completion of treatment and earlier patient discharge. A wide range of injuries and conditions can thus be managed safely, efficiently, and in a resource-conserving manner under pRA in the ED, without the need to involve OR capacities. Compared with LA, pRA allows the treatment of larger areas, including the use of a tourniquet (Figure 1), provided that standard basic monitoring (non-invasive blood pressure, ECG, and pulse oximetry) is maintained in accordance with safety recommendations [6]. By contrast, PS and GA share comparable risk profiles, necessitating monitoring and prolonged post-procedural observation [7]. Traditionally, the administration of pRA has been the responsibility of anesthesiologists, given the methodological demands and potential side effects or complications, and should be performed in an anesthetic setting with the corresponding facilities [8].

With the introduction of ultrasound (US) as an imaging modality enabling controlled and targeted real-time injection of local anesthetics, the success rates, safety, and onset times of pRA have significantly improved [9]. However, a safe and reliable application requires ED physicians to be trained in US-guided pRA techniques. In recent years, several emergency medicine societies have incorporated ultrasound-guided peripheral nerve block techniques into their core training curricula and competency frameworks, emphasizing their growing relevance for routine ED practice [10,11].

This retrospective single-center analysis reports on the management of acute surgical injuries, dislocations, and conditions using US-guided pRA in the orthopedic ED of an affiliated hospital (Merian Iselin Klinik, Basel, Switzerland). The primary aim was to evaluate the feasibility, safety, and effectiveness of this approach over time. This study covers a six-year period and focuses on procedures performed outside of the operating theater setting in a cohort of 1117 patients.

## 2. Materials and Methods

### 2.1. Patient Selection

During the observation period, only cardiopulmonarily stable and fully conscious patients were included for further treatment.

**Figure 2:**
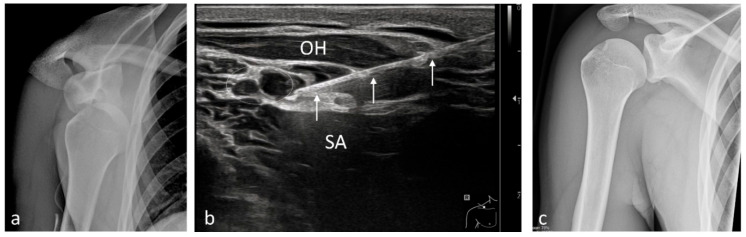
Reduction of an anteroinferior right-sided (R) shoulder dislocation under pRA. (**a**) Anteroposterior (AP) radiograph prior to reduction. (**b**) The three white arrows indicate the needle advancing beneath the superior trunk (white dotted oval) between the omohyoid muscle (OH) and the serratus anterior muscle (SA). (**c**) AP radiograph after correction of the dislocation.

**Figure 3:**
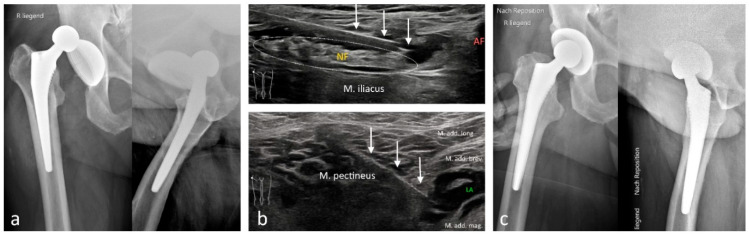
Reduction of a posterior hip total prosthesis dislocation under pRA. (**a**) Anteroposterior (AP) and axial radiographs prior to reduction. (**b**) **Top**: The three white arrows indicate the needle over the femoral nerve (NF, white dotted oval) above the iliacus muscle; the femoral artery (AF) is partially visible. **Bottom**: Anesthesia of the obturator nerve. The three white arrows indicate the needle during injection of local anesthetic between the adductor magnus and adductor brevis muscles. (**c**) AP and axial radiographs after reduction.

**Figure 4:**
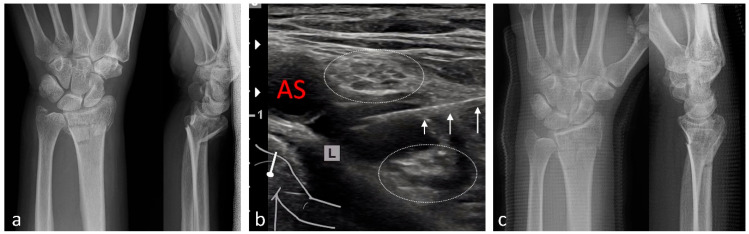
Reduction of a distal left-sided (L) radius fracture under pRA. (**a**) Anteroposterior (AP) and lateral radiographs prior to reduction. (**b**) The three white arrows indicate the needle during infraclavicular advancement and injection of local anesthetic between the lateral and medial fascicles of the brachial plexus (white dotted ovals). AS: subclavian artery. (**c**) AP and lateral radiographs after ultrasound-guided reduction in the fracture and application of a plaster splint in the ED.

**Figure 5:**
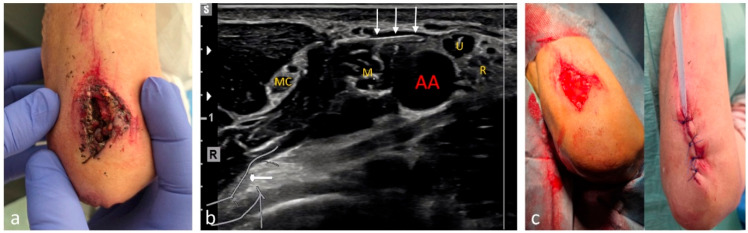
Management of a deep and heavily contaminated laceration-crush injury over the olecranon under pRA. (**a**) Pre-operative situation: remnants of soil and vegetation are visible extending down to the periosteum of the ulna. (**b**) Axillary brachial plexus block. The three white arrows indicate the needle advancing toward the ulnar nerve (U), which in this region lies in close proximity to the axillary artery (AA) together with the median (M) and radial (R) nerves. The musculocutaneous nerve (MC) has already entered between the biceps brachii and coracobrachialis muscles. (**c**) Intraoperative and post-debridement situation in the ED.

**Figure 6:**
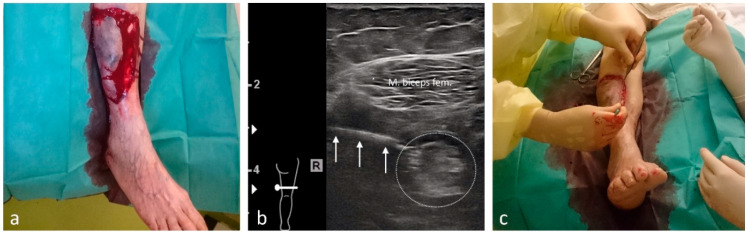
Management of a large pretibial degloving right-sided (R) injury under pRA. (**a**) Extensive degloving injury of the right lower leg in a 91-year-old patient. (**b**) The three white arrows indicate the lateral advancement of the needle between the vastus lateralis and biceps femoris muscles for a distal sciatic nerve block (white dotted oval). The local anesthetic is shown surrounding the nerve. (**c**) Intraoperative situation during management of the degloving injury in the ED.

**Figure 7:**
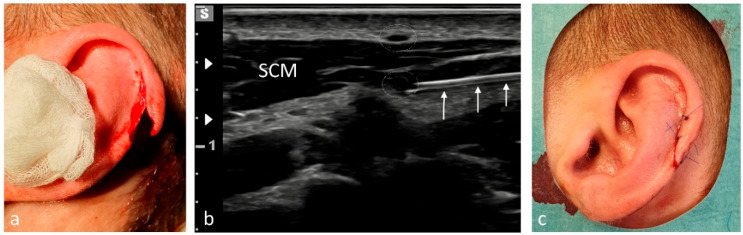
Anesthesia of the auricle for treatment of a traumatic helix laceration under pRA. (**a**) Laceration of skin and cartilage following a head impact during soccer. (**b**) The three white arrows indicate the needle advancing beneath the sternocleidomastoid muscle (SCM), where the internal branch of the great auricular nerve (GAN, white dotted oval) is located. The external branch of the GAN (white dotted oval) appears above the SCM after curving around its posterior border. (**c**) Post-procedural situation after repairing the helix.

**Figure 8:**
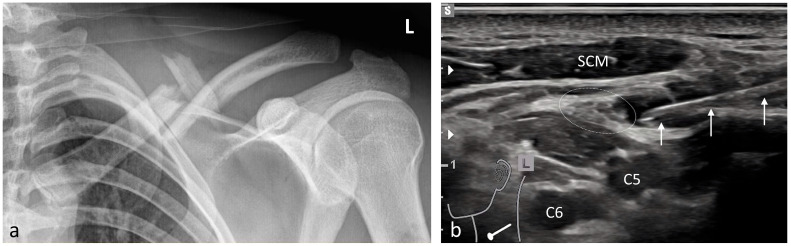
Analgesia of a severely painful multi-fragmentary left-sided (L) clavicle fracture using pRA. (**a**) Anteroposterior (AP) radiograph of the clavicle. (**b**) The three white arrows indicate the needle during injection of local anesthetic around the supraclavicular nerves (white dotted oval), which at this level are located above the prevertebral fascia. Beneath the fascia, the ventral rami of the C5 and C6 spinal nerves are situated. SCM: sternocleidomastoid muscle.

Simple pain syndromes—such as sprains not requiring surgical or orthopedic intervention—were treated with enteral or parenteral analgesia. Minor localized injuries were managed under LA. US-guided pRA was offered as an alternative to PS or GA in the following situations:

- Short interventions such as reductions (Figure 2, Figure 3 and Figure 4);
- Treatment of more extensive extremity injuries that could be managed directly in the ED as an alternative to operative management (Figure 5 and Figure 6);
- Superficial injuries in the head–neck region, if an epifascial block was feasible (Figure 7);
- Painful clavicular fractures, if a purely epifascial blockade of the supraclavicular nerves was possible (Figure 8);
- Acute exacerbations of pain syndromes as initial analgesic treatment.

Patients who declined pRA or who required immediate surgical intervention (e.g., osteosynthesis or deep subfascial soft-tissue injuries) initially received systemic analgesia and were subsequently referred to the orthopedic or trauma specialists for further care. These patients were admitted for inpatient treatment and managed by anesthesiologists and orthopedic surgeons. GA was not performed in the ED.

### 2.2. Contraindications

Absolute contraindications for pRA were patient refusal, known allergies or intolerance to the applied local anesthetics, and local infection at the intended puncture site.

In patients receiving anticoagulant therapy, only superficial peripheral nerve blocks without proximity to major vascular structures were performed. Deep blocks, such as the psoas compartment block, were avoided in accordance with current safety guidelines.

### 2.3. Structure and Staffing of the Emergency Department

The clinic operates as an affiliated hospital with specialties in urology, orthopedics, surgery, otorhinolaryngology, sports medicine, and internal medicine, and provides a wide range of medical and surgical services. A total of 110 inpatient beds are available.

The ED, with approximately 6500 visits annually, functions as an independent department in addition to anesthesiology. It is responsible for the interdisciplinary management of all non-life-threatening conditions. Patients with acute life-threatening conditions are stabilized and transferred to nearby tertiary centers.

The ED staff includes physicians from various specialties at both consultant and resident levels. During the study period, a total of 31 physicians worked in the ED, including 5 consultants in anesthesiology, 6 consultants in orthopedics, 5 consultants in surgery, 3 consultants in internal medicine, 1 consultant in general medicine and 11 residents in training. The average duration of employment was 17 months (range 3–72 months). Sixteen physicians (52%) performed a pRA. The ED operated daily from 08:00 to 22:00 in a two-shift model. Staffing included eight positions, ensuring continuous coverage by at least two physicians, one of whom was board-certified. Duty scheduling was independent of specialty. When required, the head of the ED (ES) or their deputy was available for consultation outside regular hours. All ED physicians completed a general emergency ultrasound training course at the beginning of their employment, as well as a certified two-day basic course in US-guided anesthetic interventions. Additionally, a 30 min weekly training session was held to standardize and reinforce safe US-guided pRA practice for both medical and nursing staff.

Special emphasis was placed on consistent adherence to three safety principles:

- Reliable identification of the target structure and potentially vulnerable adjacent structures (e.g., vessels, pleura);
- No advancement of the needle unless the tip is clearly visualized;
- No further injection of local anesthetic unless a typical sonographic distribution pattern was evident.

### 2.4. Peripheral Regional Anesthesia

In the ED, only pRA techniques were applied, in which, unlike neuraxial techniques, no systemic complications or side effects were expected. All pRA procedures were performed under ultrasound (US) guidance in accordance with institutional standards. Two high-resolution US systems (Samsung RS80 EVO (Seoul, Republic of Korea) and Canon Aplio a (Tokio, Japan)) with appropriate linear probes (Samsung LA4-18B (Seoul, Republic of Korea), Canon PLT-1204BT (Tokio, Japan) were available.

All US-guided pRAs in the ED were performed by attending emergency physicians and senior orthopedic residents who had completed a structured US-guided pRA training pathway led by the first author, an anesthesiologist and certified course director in pRA. An anesthesiologist was on site in the hospital at all times but not routinely present during ED pRA; immediate consultation was available if required.

Each pRA was performed after establishing intravenous access and under standard basic monitoring (non-invasive blood pressure, ECG, and pulse oximetry). Exceptions included blocks of small peripheral nerves of the forearm, hand, or foot, which—due to the very low anesthetic volumes used—were occasionally performed without IV access or continuous monitoring, in accordance with institutional safety standards.

In outpatients, only single-shot techniques were applied. For inpatients, catheters for continuous anesthetic infusion were placed as needed by the anesthesiology team. All procedures were conducted under strict sterile conditions, including sterile draping of the intervention area and ultrasound probe, and the use of sterile US gel. Echogenic puncture needles were exclusively employed (PAJUNK (Geisingen, Germany) SonoBlock 20 G × 120 mm, 20 G × 50 mm; PAJUNK (Geisingen, Germany) SonoTAP 24G × 40 mm; TRANSMED (Felsberg, Germany) Reganesth 22G × 80 mm).

Local anesthetics used:

- Prilocaine 2% for short-acting blocks;
- Ropivacaine 0.75% or 1%, optionally combined with clonidine 75 µg for longer-lasting effects (e.g., exacerbated pain syndromes).

Applied volumes were adapted to the specific block technique (see Table 1). In cases of proximal tibial fractures, a femoral nerve block was selected for analgesia. This approach is anatomically justified, as the femoral nerve provides sensory innervation to the anterior knee capsule and the quadriceps insertion at the tibial head (Hilton’s law).

Each pRA was documented in the clinical information system (M-KIS, Meierhofer AG) with details on indication, technique, efficacy, and potential adverse events or complications. Additionally, imaging documentation was stored as a video clip or still image in the hospital’s picture archiving and communication system.

To ensure patient safety, resuscitation equipment, intralipid emulsion for the treatment of local anesthetic systemic toxicity (LAST), and emergency drugs were immediately available in the ED at all times. All physicians involved were trained in the recognition and management of LAST according to current guidelines.

### 2.5. Data Collection and Statistical Analysis

Procedures were documented using the terms “ultrasound-guided” or “sonographically guided” and “regional anesthesia”, with exact specification of the block site and targeted nerves. Based on this terminology, the database was searched and analyzed.

Procedures performed under LA (e.g., minor wound management) or sonographically assisted interventions such as joint aspiration, abscess drainage, or cyst aspiration were excluded. The remaining datasets were analyzed for diagnosis groups, applied pRA techniques, and treatment. Block success as well as complications or side effects were also evaluated. Statistical analysis was descriptive and performed using Microsoft^®^ Excel^®^ 365 MSO (Version 2506, Build 16.0.18925.20076), 64-bit edition.

## 3. Results

Between February 2018 and January 2024, a total of 37,609 patients were treated in the ED. Of these, 36,959 patients (male *n* = 17,510; female *n* = 19,449; mean age 50.9 years [range 2–103]) presented with emergency-related conditions only. Among them, 1516 cases were non-orthopedic diagnoses, resulting in 35,443 orthopedic-trauma emergency patients within the study period. A total of 33,748 patients (95.2%) were managed on an outpatient basis in the ED. Of the orthopedic-trauma patients, 1292 (3.7%) underwent either US-guided pRA (*n* = 1117; 3.2%) or PS (*n* = 175; 0.5%) for treatment.

Among the 1117 pRA patients, 249 (22%) received the block for extensive wound management or reduction of dislocations (Figure 1, Figure 2, Figure 3, Figure 4, Figure 5, Figure 6 and Figure 7). In the remaining 868 cases (78%), pRA was applied for analgesia only, for example, to facilitate further diagnostic evaluation and immobilization of non-immediately operable fractures, or in cases of lumbago, cervical pain, or arthralgia. No complications related to pRA were observed (Table 1). In 12 of the 175 PS patients, a contraindication to pRA was present. Thirteen patients (0.1%) declined pRA, and in 3 cases, pRA was not performed due to lack of compliance. In 147 patients requiring reduction, a pRA could have been performed, but was not administered by the attending ED physicians; these patients were instead managed with PS.

## 4. Discussion

In this six-year single-center analysis, US-guided pRA performed directly in the orthopedic ED proved to be a safe and effective approach for managing selected non-life-threatening traumatic and pain-related conditions. The use of pRA enabled timely and definitive treatment within the ED, avoiding the need for PS or GA and thereby preserving anesthetic and surgical resources.

The absence of pRA-related complications and the high procedural success rate in more than one thousand patients underscore the fact that, when performed under structured protocols and adequate US-training, pRA can be reliably and safely integrated into routine ED practice. These findings further support the growing international trend toward the implementation of US-guided pRA programs within EDs as a cornerstone of multimodal and resource-efficient pain management.

This feasibility was mainly attributable to the fact that, in most cases, the underlying diagnoses allowed treatment outside the OR. These primarily included reductions in fractures and dislocations, as well as extensive wound management procedures that required urgent intervention but no deeper surgical exploration in the OR. By enabling immediate care within the ED, both the fully utilized OR capacities and the limited anesthetic resources could be effectively preserved [12].

A key advantage of pRA is its excellent analgesic effect while maintaining consciousness. This avoids typical side effects of GA or PS, such as postoperative cognitive dysfunction in elderly patients, and, in particular, respiratory and hemodynamic complications [7,13]. Although such complications are generally uncommon (<10%), they require prolonged monitoring or specific therapeutic measures in individual cases.

The same applies to neuraxial techniques such as spinal or epidural anesthesia, which also require extended monitoring and observation and were therefore not applied in this setting [14].

For PS or GA, especially in non-vital indications, higher safety requirements must be met, including fasting status and continuous monitoring of vital signs. Moreover, in patients with significant cardiopulmonary or endocrine comorbidities, the systemic effects of PS or GA necessitate a more complex approach, which would have exceeded the ED’s capacities. In contrast, the safe application of pRA has been well documented even in high-risk patients [15,16,17].

Two decades ago, pRA was rarely used in the ED. Since then, its application has steadily increased, paralleled by a growing number of publications [10]. The effectiveness and safety of pRA are now also well established in emergency medicine, and pRA is increasingly being adopted by emergency physicians as the primary modality for severe pain management [11,12,18].

In many trauma situations, rapid pRA is indicated even before radiological diagnostics, particularly in proximal femoral fractures or joint dislocations. Radiological imaging required for treatment planning should not delay the immediate initiation of effective analgesia. In such cases, fracture confirmation or exclusion may be achieved sonographically during nerve blockade, enabling immediate pain relief without compromising diagnostic accuracy [19,20].

Across the entire ED patient population, however, pRA represented the appropriate method only for a minority of cases. During the six-year observation period, 3.2% of all orthopedic patients were managed exclusively under pRA. This proportion remained largely stable throughout the study period. This constancy is primarily explained by the fact that most patients were sufficiently managed with conservative enteral or parenteral analgesia, and pRA was required only for definitive treatment or in cases of exacerbated pain.

Nevertheless, as not all patients are suitable for pRA, comprehensive counseling and consideration of individual factors are essential. In the present cohort, only 13 patients (1%) refused pRA after counseling and insisted on GA or PS. In 3 cases, pRA was not performed due to a lack of compliance. Importantly, GA was avoided in all cases, resulting in a significant reduction in anesthetic resource utilization.

The use of landmark-guided pRA for targeted, low-risk analgesia in proximal femoral fractures in the ED was first reported in 1995 [21], and for over 15 years, US-guidance has been established [22]. In this analysis, femoral nerve block (25%) was, alongside brachial plexus blockade (54%) and distal forearm nerve blocks [23,24,25], the most frequently applied pRA technique for extremity injuries. In proximal femoral fractures and hip arthroplasty dislocations, combined blocks of the lumbar plexus nerves were required (27%).

In recent years, US-guided fascial blocks in the ED have increasingly been reported at various anatomical sites [10,18,26]. These techniques act primarily via the injection of high volumes of local anesthetic into fascial compartments and are relatively non-specific. Compared to targeted nerve or plexus blocks, they are less precise and, due to the larger anesthetic volumes, act more systemically [27]. Consequently, side effects are possible, limiting the advantages of pRA. For this reason, such techniques were used in the present cohort only in patients with exacerbated lumbago, where targeted pRA was not feasible (6.1%). Specifically, these were erector spinae plane blocks, which primarily anesthetize the intramuscularly located dorsal rami rather than, as often assumed, the entire spinal nerve. The latter was achieved via paravertebral blocks in patients with rib fractures (*n* = 17; 1.5%). Since the risk profile of paravertebral blocks is comparable to classical neuraxial techniques, they were exclusively performed by ED staff with anesthesiology training.

The broad use of pRA in EDs remains limited by several factors. First, the effectiveness of traditional landmark techniques is not always reliable. Second, concerns persist regarding potential side effects or complications, which may include puncture-related complications (hematomas, pneumothorax, nerve injury) and systemic local anesthetic toxicity requiring inpatient or even intensive care management. Since the introduction of US, however, block success rates have markedly improved with correct indication and technique [6]. Anatomical variants and vulnerable structures can be visualized, and misplacement can be avoided. With real-time US-guidance and targeted injection of significantly reduced anesthetic volumes, unintended spread and systemic side effects can largely be prevented [6,28,29,30,31].

The local anesthetic doses used in this study were below standard regimens and corresponded to the low-dose protocols described in the literature [32,33,34,35]. For single-nerve blocks, volumes were limited to only 2–4 mL, at which toxic effects are not expected [36]. Such low dosages were feasible due to real-time visualization, targeted injection, and immediate adjustment of needle position when distribution was not optimal.

It must be noted that the distribution of local anesthetics in preformed anatomical compartments follows pressure gradients. The frequently cited “vascular-nerve sheaths,” enabling uniform spread along nerve pathways, are anatomically unproven and rather theoretical constructs [37]. In reality, peripheral nerves and vessels run within compartments delineated by muscle and fascial layers, filled with loose connective and adipose tissue. Due to their common embryologic origin, nerves are consistently located in close proximity to arteries and veins. Furthermore, anatomical barriers such as muscular connections (e.g., between the scalene muscles) can impede uniform distribution of local anesthetics and thus prevent complete anesthesia of all nerve components [31,38]. The effective lumen of anatomical compartments is often smaller than expected, so larger anesthetic volumes follow local pressure gradients, making spread less predictable [39].

Except for selected erector spinae and paravertebral blocks, the patients were primarily managed with peripheral nerve and plexus blocks. When performed with correct indication and technical accuracy, these provide excellent analgesia with high safety. Nevertheless, pRA techniques are technically demanding and require profound knowledge of sonoanatomy and strong ultrasound skills. This study demonstrated that, despite basic pRA training, some staff members lacked the advanced sonographic or anesthetic expertise necessary in specific cases.

A Canadian survey of emergency physicians found that the main barrier to pRA was not lack of equipment, but rather insufficient training, a deficit that significantly limits clinical implementation [40]. Nevertheless, there is broad consensus in the literature that ultrasound-guided pRA in the emergency setting can greatly improve patient comfort and safety, including faster onset, higher success rates, shorter procedure times, and the ability to use lower local anesthetic doses [23].

### 4.1. Training

The goal of ED physician training was the precise performance of ultrasound-guided pRA techniques to maximize efficacy and safety while minimizing anesthetic dosage. All physicians, therefore, completed a certified course in ultrasound-guided interventions and vascular access, supplemented by regular training on technique, pharmacology, and complication management. Independent practice was supervised by the ED director (ES). However, it became evident that initial training alone was insufficient without immediate, hands-on implementation. Safe application of US-guided pRA requires not only theoretical knowledge but also ongoing, guided practical experience. Of the 175 PS cases, the majority—including 79 shoulder dislocation reductions—could have been managed under pRA instead. The main barrier was a lack of confidence among ED physicians in applying the specific techniques [41]. Therefore, these advanced procedures should be integrated into training curricula and incorporated into routine clinical practice.

The scientific and clinical background of ED personnel is another key determinant for the successful implementation of pRA. In the present setting, most emergency physicians were trained primarily in orthopedics or traumatology, with limited formal anesthesiology exposure. While this facilitated rapid procedural decision-making and familiarity with musculoskeletal pathology, it also emphasized the need for structured anesthetic training and continuous interdisciplinary collaboration to maintain the high safety standards required for pRA.

### 4.2. Practical Implications and Recommendations for Implementing US-Guided pRA in the ED

The present analysis highlights several practical aspects relevant for the establishment of US-guided pRA programs in emergency departments.

1. Program structure and governance: Implementation should be organized as a structured service, ideally supervised by a designated pRA lead with expertise in both anesthesia and emergency medicine. Clear standard operating procedures (SOPs) should define indications, contraindications, monitoring, documentation, and management of local anesthetic systemic toxicity (LAST).
2. Training and competency: Successful integration of pRA requires systematic training. Initial courses in US-guided vascular access and peripheral nerve blocks should be complemented by supervised hands-on sessions and continuous quality assurance. Regular refresher training and logbook documentation help to maintain Iprocedural safety and confidence.
3. Safety and monitoring: Even though pRA carries a low systemic risk, standardized safety measures are essential. Monitoring should at least include non-invasive blood pressure, ECG, and pulse oximetry, with intravenous access established before block placement. A local protocol for immediate management of potential complications, including lipid rescue therapy, should be available in every ED.
4. Workflow and resource optimization: The introduction of pRA can significantly reduce the need for PS or GA in the ED. To maximize efficiency, ready-to-use pRA trays, sterile US equipment, and designated treatment areas should be provided. Early identification of suitable cases—such as proximal femur fractures, shoulder or wrist dislocations, or large wound management—facilitates timely analgesia and faster throughput.
5. Evaluation and quality metrics: Continuous data collection on indications, block success, time to analgesia, conversion to PS or GA, and complications are essential for benchmarking and further improvement. Regular morbidity and quality meetings strengthen safety culture and interdisciplinary collaboration.

These recommendations align with current literature demonstrating that structured training, safety protocols, and interdepartmental collaboration are key determinants of successful and sustainable US-guided pRA programs in the ED setting [42,43].

## 5. Conclusions

Ultrasound (US)-guided peripheral regional anesthesia (pRA) represents a safe and effective alternative to procedural sedation (PS) or general anesthesia (GA) for selected non-life-threatening orthopedic emergencies in the ED. Its implementation enabled timely and definitive treatment while preserving anesthetic and surgical resources and avoiding prolonged monitoring or inpatient admission. The findings of this study confirm the high safety profile of pRA when performed under structured protocols and adequate ultrasound training. Consequently, US-guided pRA should be incorporated as an essential component of emergency physician education and ED service development.

## Figures and Tables

**Table 1:** Summarizes the diagnoses and the pRA techniques used for the corresponding indications.

Fracture reductions and immobilizations (*n* = 644)		
**Indication/Diagnosis**	***n***	**Target Nerves/pRA Technique (Local Anesthetic Volume in mL)**
Clavicle	48	Supraclavicular nerves/2–3
Scapula	8	Brachial plexus block (interscalene)/3–5
Humerus	148	Brachial plexus block (supraclavicular)/10 (5)–20
Olecranon	8	Brachial plexus block (infraclavicular)/10–20
Forearm (complete)	24	Brachial plexus block (axillary)/12–20
Radius	111	Brachial plexus block (infraclavicular)/10–20
Hand	6	Hand block (ulnar, median, superficial radial)/2–3 per nerve
Proximal femur fractures	188	Femoral and obturator nerves/10 each
Acetabulum	2	Psoas compartment block/20
Femoral shaft/distal femur	25	Femoral and obturator nerves/10 each
Patella	7	Femoral nerve/10
Tibial head	12	Femoral nerve/10
Lower leg	3	Femoral and sciatic nerves/10 each
Ankle and foot	34	Femoral and sciatic nerves/10 each
Pelvis	3	Erector spinae block/7–10
Ribs	17	Paravertebral block/4–10
Dislocation reductions and immobilizations (*n* = 174)		
**Indication/Diagnosis**	***n***	**Target Nerves/pRA Technique (Local Anesthetic Volume in mL)**
Shoulder	99	Brachial plexus block (interscalene)/3–5
Shoulder prosthesis	5	Brachial plexus block (interscalene)/3–5
AC joint	11	Supraclavicular nerves/2–3
Elbow	13	Brachial plexus block (infraclavicular)/15–20
Wrist	1	Brachial plexus block (infraclavicular)/15–20
Hip prosthesis	21	Femoral and obturator nerves/10 each
Knee prosthesis	3	Femoral nerve/10
Patella	2	Femoral nerve/10
Ankle	11	Sciatic and saphenous nerves/10 each
Fingers	8	Hand block (ulnar, median, superficial radial)/2–3 per nerve
Analgesia/treatment of soft tissue lesions (*n* = 150)		
**Indication/Diagnosis**	***n***	**Target Nerves/pRA Technique (Local Anesthetic Volume in mL)**
Foreign body removal	14	Depending on location
Hematomas and hemarthroses	15	Depending on location
Soft-tissue infections (extremities)	46	Depending on location
Large lacerations/cuts	12	Depending on location
Rotator cuff pathology	63	Brachial plexus block (interscalene)/3–5
Cervicalgia/Lumbago (*n* = 114)		
**Indication/Diagnosis**	***n***	**Target Nerves/pRA Technique (Local Anesthetic Volume in mL)**
With radiculopathy	80	Cervicalgia: target nerve depending on region; Lumbago: erector spinae/7–20
Without radiculopathy	34	
Pain-exacerbated arthropathies (*n* = 35)		
**Indication/Diagnosis**	***n***	**Target Nerves/pRA Technique (Local Anesthetic Volume in mL)**
Shoulder osteoarthritis	19	Brachial plexus block (supraclavicular)/10 (5)–20
Other arthralgia	16	Depending on location

## Data Availability

Data supporting the reported results can be found in the clinical information system (M-KIS, Meierhofer AG) of Merian Iselin Klinik Basel.

## Abbreviations

The following abbreviations are used in this manuscript:

US	Ultrasound-guided
pRA	Peripheral regional anesthesia
GA	General anesthesia
PS	Procedural sedation
ED	Emergency department
LA	Local anesthesia
